# The Role of Campus Data in Representing Depression Among College Students: Exploratory Research

**DOI:** 10.2196/12503

**Published:** 2020-01-27

**Authors:** Guang Mei, Weisheng Xu, Li Li, Zhen Zhao, Hao Li, Wenqing Liu, Yueming Jiao

**Affiliations:** 1 Department of Control Science and Engineering College of Electronic and Information Engineering Tongji University Shanghai China; 2 Informatization Office Tongji University Shanghai China

**Keywords:** depression, mental health, behavior analysis

## Abstract

**Background:**

Depression is a predominant feature of many psychological problems leading to extreme behaviors and, in some cases, suicide. Campus information systems keep detailed and reliable student behavioral data; however, whether these data can reflect depression and we know the differences in behavior between depressive and nondepressive students are still research problems.

**Objective:**

The purpose of this paper is to investigate the behavioral patterns of depressed students by using multisource campus data and exploring the link between behavioral preferences and depressive symptoms. The campus data described in this paper include basic personal information, academic performance, poverty subsidy, consumption habit, daily routine, library behavior, and meal habit, totaling 121 features.

**Methods:**

To identify potentially depressive students, we developed an online questionnaire system based on a standard psychometric instrument, the Zung Self-Rating Depression Scale (SDS). To explore the differences in behavior of depressive and nondepressive students, the Mann-Whitney U test was applied. In order to investigate the behavioral features of different depressive symptoms, factor analysis was used to divide the questionnaire items into different symptom groups and then correlation analysis was employed to study the extrinsic characteristics of each depressive symptom.

**Results:**

The correlation between these factors and the features were computed. The results indicated that there were 25 features correlated with either 4 factors or SDS score. The statistical results indicated that depressive students were more likely to fail exams, have poor meal habits, have increased night activities and decreased morning activities, and engage less in social activities (eg, avoiding meal times with friends). Correlation analysis showed that the somatic factor 2 (F4) was negatively correlated with the number of library visits (*r*=–.179, *P*<.001), and, compared with other factors, had the greatest impact on students’ daily schedule, eating and social habits. The biggest influencing factor to poor academic performance was cognitive factor F1, and its score was found to be significantly positively correlated with fail rate (*r*=.185, *P*=.02).

**Conclusions:**

The results presented in this study indicate that campus data can reflect depression and its symptoms. By collecting a large amount of questionnaire data and combining machine learning algorithms, it is possible to realize an identification method of depression and depressive symptoms based on campus data.

## Introduction

Depression is a serious mental health issue that affects a significant segment of the population and has become a leading cause of disability and suicide. It is estimated that nearly 300 million people suffer from depression [1]. The best time to address this health problem is before symptoms disrupt patients’ daily life—for example, early warning of mood changes and anxiety can dramatically reduce chronic psychological distress and loss of function [2-4]. Analyses of behavior, sentiments, and other outward signs of depression are of great importance to the diagnosis and treatment of patients.

As students suffer from pressure stemming from financial status, academic demand, interpersonal relationship [5], and career [6], college campuses are among the environments hardest hit by depression. Depression leads to poor academic performance [7,8], drinking problems [9], suicidal thoughts [10,11], frequent illness [12], and dropout [13] and is prevalent in many countries [14-19]. According to a survey reported by the US Centers for Disease Control and Prevention in 2017, 31.5% of US students nationwide reported feeling so sad and hopeless almost every day that they stopped doing some usual activities [20]. Similar phenomena were discovered by the American College Health Association after they studied 80,121 students from 106 schools in 2009 [21]: 43% of the respondents reported that at least once within the previous school year they “felt so depressed that it was difficult to function,” and more than 62% “felt hopeless.” Moreover, the proportion of depressive students has been reported to have increased rapidly during recent years [6] by many universities and associations.

The situation in Chinese colleges is far from optimistic as well. In a review of 39 studies including 32,694 university students, Lei et al [22] indicated that the overall prevalence of depression among Chinese students was 23.8%, highlighting the urgent need to tackle the issue.

At present, mental health services provided on college campuses are inadequate. Students taking the initiative to ask for help and report their symptoms are the first indication for the deployment of mental health counseling services; however, the proportion of students who actively reported psychological problems was only 18% [23]. A promising approach is to identify useful clinical behavior indicators and use them to estimate the occurrence of depression.

Behavioral models of depression suggest that decreased response-contingent positive reinforcement for previously rewarded behaviors is the central fact for bringing about depression [24]. Decreased positive reinforcement may be caused by changes in the quantitative or qualitative aspects (eg, social, intellectual; function: stimulation seeking, achievement) of the reinforcing events, availability of reinforcement in the environment (eg, social isolation, poverty), inadequate instrumental behaviors (eg, social skill, academic ability), and/or the result of an increased frequency of punishment [25]. The theory implies the possibility that some external characteristics may explain or lead to depression. In the case of a university campus, data that can reflect these characteristics include basic personal information, academic performance, poverty subsidy, consumption, daily routine, library use, meal habit, sport, club activity, etc.

Depression is a common disorder that impacts an individual’s ability to perform life activities, including those required by academic life. The significant negative relationship between depression and academic performance was identified by many researchers [26,27], showing that students suffering from moderate levels of depression demonstrated lower performance compared with those with normal and mild levels of depression [26]. Reading preference as an important factor contributing to academic performance was also found to correlate with depression [28].

Food and eating habits have an impact on the development of depression, and depression can, in turn, affect the patient’s eating routine [29]. A study on dietary habits and food intake in adults aged 50 years and older revealed that people in the depressed group had a poor appetite, almost never dined out, and ate alone [29]. Vice versa, altered eating behaviors showed elevated levels of impulsivity and depression [30].

Social relationships play a key role in depression. One typical feature of the disorder is social isolation and social withdrawal [31]. Another core characteristic is anhedonia—loss of interest or pleasure in previously enjoyable activities [32]. Depressed patients tend to opt out of social situations, either formally (eg, exiting an art group) or informally (eg, being unwilling to see friends). Moreover, for most depressed patients, their social connections have already been significantly reduced prior to the development of depression symptoms. Therefore, the reduction of social connections is a key feature of depression, and this is more prevalent than in other physical and mental illnesses [33]. Cacioppo et al [34] found that even after controlling for key candidate variables such as demographics, personality, physical health, stress, and many factors related to social relationships, perceived social isolation is a good longitudinal predictor of depressive symptoms.

Mood disorder can be considered a biorhythm disorder [35]. The symptomatology of depression implies that a biological clock disorder may result in the occurrence of depression [35]. This is also why depression is associated with sleep disorders. For example, the time between falling asleep and the first rapid eye movement sleep in a depressed patient is much shorter than that of a nondepressive person [36]. In addition to being manifested in sleep disorders, biorhythm disorders can also cause disorders in other physiological mechanisms. For people with depression, the severity of mood and other symptoms also fluctuate during the day [37]. They feel worse in the morning, and the situation is slightly better during the day. Other biological rhythms, such as body temperature and cortisol level, also become irregular [36]. On the other hand, depressed persons have their own activity preferences. By investigating a sample of 400 undergraduate students, Sheslow et al [38] found that nondepressive students tended to eat with others and engage with others more often than depressive students, and they implied that depression in the university population is correlated with small changes in a large number of daily activities. However, there is currently no literature on the relationship between depression and its symptoms and daily routine preferences of college students.

It is worth noting that depression consists of a variety of symptoms. In most studies that use the Zung Self-Rating Depression Scale (SDS) for measuring levels of depression, the total score was used as an indicator of the severity of depression; however, overreliance on the total score of the SDS is undesirable [39] and may not be able to identify different types of depression because various profiles of heterogeneous symptoms are included in a single dimension of severity. For example, the total score cannot distinguish between those who are mainly suffering from physical symptoms and those who are mainly affected by affective symptoms [40]. It is necessary, therefore, to study the related influencing elements of various symptoms of depression.

As summarized above, there are two main shortcomings of current body of literature with regard to the subject of college student depression. First, there is a lack of research on the relation between college students’ depression and their behavior, essential for a better understanding of the behavior pattern of the affected. Second, previous studies were mainly based on questionnaire surveys, which may bring about the pervasive problem of social desirability bias [41].

## Methods

### Questionnaire

The SDS [42] was used as the primary tool to determine the depression levels of college students. It is a 20-item Likert-type instrument (4-point scale) to measure depressive affect and related symptomatology during a week by seeking responses to questions such as “I feel downhearted and blue,” “Morning is when I feel the best,” and “I have crying spells or feel like crying.” Participants were asked to choose one of the following responses to each of the questions: 1=rarely or none of the time (<1 day), 2=some or a little of the time (1-2 days), 3=occasionally or a moderate amount of the time (3-4 days), 4=most or all of the time (5-7 days).

SDS is one of the most common screening tests used by clinicians and psychiatrists. Participants’ raw scores ranged from 20 to 80. The higher the scores, the higher the occurrence of symptomatology. The standard point of the SDS is obtained by multiplying the raw score by 1.25. According to the Chinese norm for the general public, scores of 53 to 62 are classified as mild depression, 63 to 72 moderate depression, and 72 to 100 severe depression. SDS has an adequate item homogeneity; Knight et al [43] reported an alpha coefficient of .79 in a large community sample, and Gabreys and Peters [44] reported an alpha value of .88 in a sample of depressed patients.

In this paper, we developed an online questionnaire system and only allowed students connected to the campus network to participate in it. In order to control the accuracy of the questionnaire results, we recorded the duration of answering and removed students who completed the form in less than 5% of the overall time distribution and used the Beck Depression Inventory (BDI) [45] as a supplementary questionnaire to detect biased answers. As conclusions drawn by SDS and BDI are strongly related to each other theoretically, we deleted very contradictory items (eg, when the SDS showed that one student suffered from moderate to severe depression while the BDI indicated no depression). We also excluded participants who never had breakfast at the college canteens because they may be off-campus students.

### Privacy Protection

We fully considered the privacy of students. First, we signed a data confidentiality agreement with the information management department and passed the ethical review of the University Department of Medical and Life Sciences (No 2018YXY24). Second, we indicated the purpose of the experiment, the type, and the time period of the use of the data on the first page of the questionnaire to let the participants know about this study.

**Figure 1:**
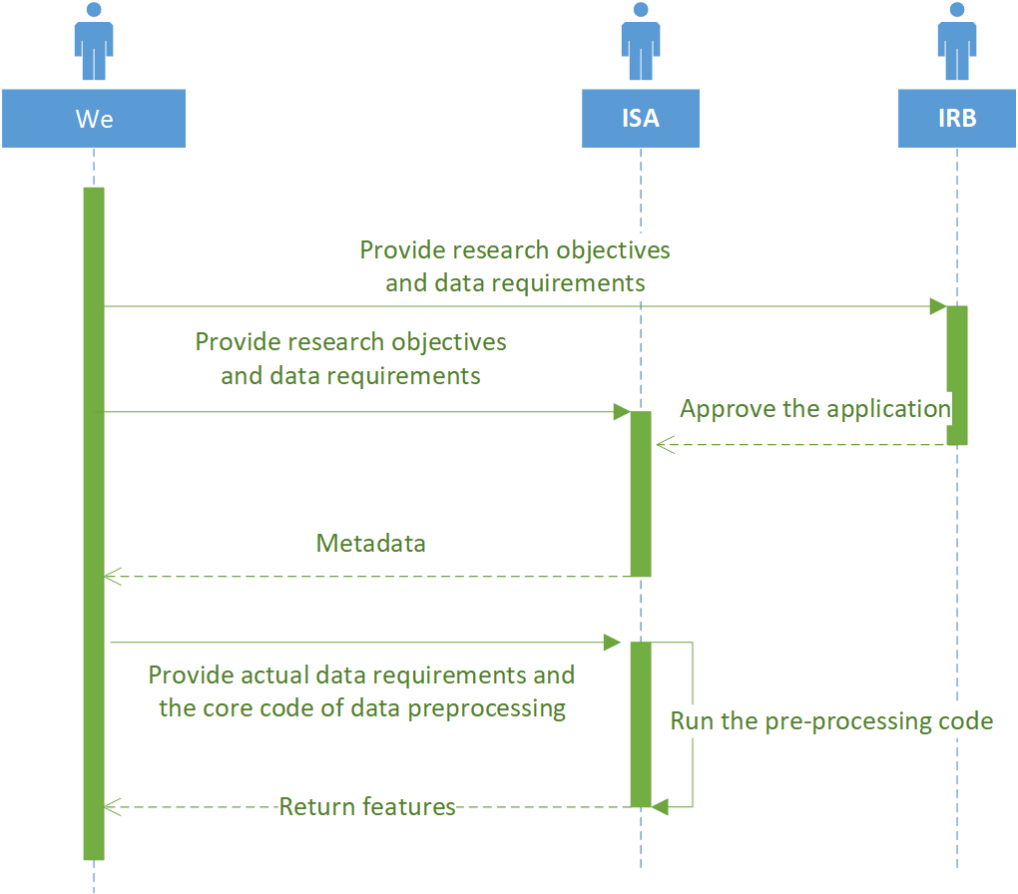
Data acquisition process.

With respect to the feature extraction, we designed a process to meet the regulations. We first gave the objectives and data requirements to the institutional review board and the information system administrator (ISA). After that, we got the metadata, such as field names with their meanings, data types, etc, of related tables from ISA. Subsequently, we provided detailed data requirements and the core code of data preprocessing. Furthermore, ISA wrote other auxiliary codes (eg, database accessing codes) and ran the whole preprocessing code on their own computers, and we finally got the features which did not contain any sensitive information such as students’ names and home addresses. In the whole process, we did not access any school information systems and therefore could not obtain any other private data. The process is illustrated in Figure 1.

### Units of Observation

The research conducted by Kessler et al [46,47] showed that depression episodes characterized as mild to severe have an average duration of 13.8 to 16.6 weeks, and very severe episodes have a mean duration of 23.1 weeks. The overall mean duration is 16 weeks. As one semester (18 weeks) is a complete cycle of student life on campus, we used data generated in the first semester of 2017 academic year (from September 11, 2017, to January 10, 2018) as the research subject for the study. The questionnaire data were collected from December 29, 2017, to January 28, 2018, and social relationship data were generated from November 20, 2017, to January 10, 2018.

### Definition of Symptoms

In order to determine the symptom groups contained in the SDS, Zung divided the SDS items into three groups based on the overt content of symptoms: pervasive affect (depressive affect and crying spells), physical equivalents (diurnal variation, sleep disturbance, decreased appetite, decreased libido, weight loss, constipation, tachycardia, and fatigue), and psychological equivalents (confusion, psychomotor retardation and agitation, hopelessness, irritability, indecisiveness, personal devaluation, emptiness, suicidal ideation, and dissatisfaction) [42]. Other literature [40,48,49] adopted factor analysis to identify common clusters across different types of people, but this is difficult because the profiles of depressive symptoms vary in different populations [48].

Although some literature proposed factor structures of college students, their conclusions may not be suitable for Chinese students because of the differences in the populations. Therefore, we redivided the SDS questionnaire into different depression symptom groups using factor analysis. The accumulated score of items that belonged to each symptom group was used to represent the severity of the symptom.

### Introduction to the Features

#### Raw Features

Most students in Chinese universities are now required to have their own student cards as identification and digital wallets to access facilities, make payments on campus, etc. Daily routines, eating behavior, and consumption behavior features were extracted by analyzing the student card events logged by the door control and consumption systems. Data generated on festival and nonfestival days were analyzed separately because students might behave differently during festivals (eg, students tend to get up late and come back late in holidays, which results in an irregular schedule). Features are summarized into 7 categories based on their characteristics and are listed in Textbox 1.

With respect to scholarship, the total amount of the award since enrolled, the amount of award obtained in the last academic year, and the number of scholarships won were obtained. Students’ exam scores were divided into 5 levels according to college tradition (excellent, good, average, pass, and fail).

**Feature categories, feature details, and numbers of features.**

Basic personal information:

- Gender (1)
- Grade (1)

Academic performance:

- Scholarship (3)
- Exam score (15)

Poverty subsidy:

- Number of applications of poverty subsidies (1)
- Applied for poverty assistance this year (1)

Consumption:

- Monthly consumption behavior (6)
- Weekly consumption behavior (4)
- Water intake at the dormitory (1)

Daily routine:

- Number of times returning to and leaving the dormitory (1)
- Number of times returning to and leaving the dormitory every hour during nonfestivals (days that are not festival days; 37)
- Number of times returning to and leaving the dormitory every hour during festivals (statutory holidays in China except for Saturday and Sunday; 32)

Library behavior:

- Number of times visiting the library (1)
- Total number of books borrowed (1)
- Number of books borrowed in the semester (1)

Meal habit:

- Standard deviation of time of breakfast during nonfestivals (1)
- Average time for breakfast during nonfestivals (1)
- Number of breakfasts during nonfestivals (1)
- Number of breakfasts during festivals (1)
- Standard deviation of time of lunch during nonfestivals (1)
- Average time for lunch during nonfestivals (1)
- Number of lunches during nonfestivals (1)
- Number of lunches during festivals (1)
- Standard deviation of time of dinner during nonfestivals (1)
- Average time for dinner during nonfestivals (1)
- Number of dinners during nonfestivals (1)
- Number of dinners during festivals (1)

#### Student Card-Based Social Features

**Figure 2:**
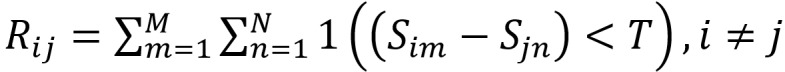
Social interaction frequency matrix.

**Figure 3:**
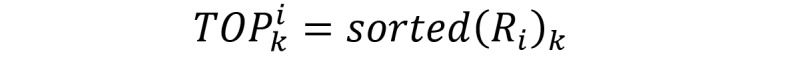
Computational method for social behavior features.

An assumption was proposed that depressed students tend to avoid meal times with their companions; if this assumption is valid, depressed students and their friends will be significantly less likely to use their student cards at the same time in the cafeteria than that of nondepressed students. Social behavior features can be defined as follows: in the equations seen in Figures 2 and 3, 1(.) is indicator function (1[true statement]=1 and 1[false statement]=0), *i* stands for the target student whose social features need to be calculated, and *j* stands for another student. M and N are swiping times of students *i* and *j*, respectively, and *S_im_* represents for the *m_th_* time when the student uses his/her student card at the campus canteens. *T* represents the time span, which was 250 seconds in this work; *R_ij_* is the social frequency between student *i* and student *j*, so the larger *R_ij_* is, the more times person *i* and person *j* shared meal times and the closer the relationship they have. *sorted(.)* is a sorting function that sorts the elements of the matrix from largest to smallest. We selected the maximum 5 values of *R_i_* as student *i*’s social features, which are named as *TOP_1_*, *TOP_2_*, *TOP_3_*, *TOP_4_*, *TOP_5_* in the following text. The effectiveness of this feature is introduced in the following section.

## Results

### Mental Health Questionnaire Results

Out of 502 undergraduate students who participated in the research, 466 students were selected as research subjects and their detailed information is shown in Table 1.

Of the participants, 69.5% (324/466) were boys and 30.5% (142/466) were girls. Ages ranged from 17 to 23 years. Grades ranged from one to six and the majority were first-year students (214/466, 45.9%). The reason there were two six-grade students in the sample is that some students repeated a grade because of various reasons (eg, receiving medical treatment).

The mean depression score of male students was 46.38, while the females’ was 49.14. The ratio of students suffering from depression was 25.32%, with mild depression (16.31%) taking the majority, and a small proportion of students were found suffering from moderate (6.44%) to severe (2.58%) depression. We used the chi-square test to determine the gender difference in depression rate, and the results are shown in Table 2.

The Mann-Whitney *U* test is a widely used nonparametric test to decide whether two groups of samples are derived from the same population. Since most of the features in this study deviated from the normal distribution, the nonparametric Mann-Whitney *U* test was used to determine whether there were differences between the two groups with respect to the behavior on campus.

The test indicated there were 25 features significantly different between depressive and nondepressive groups, and the results are listed in Table 3.

The result showed χ2=10.0 (*P*=.02), which indicated different genders had different rates of depression levels. Post hoc testing demonstrated that female students had a higher depression rate than male students (odds ratio [OR] 0.54, 95% CI 0.349-0.835); more specifically, female students had a higher risk of mild depression (OR 0.473, 95% CI 0.286-0.783), while there was no statistical difference in moderate and severe depression.

**Table 1:** Basic information of the participants.

Characteristics		Value n (%)
**Gender**		
	Female	142 (30.5)
	Male	324 (69.5)
**Age in years**		
	17	16 (3.4)
	18	170 (36.5)
	19	96 (20.6)
	20	72 (15.5)
	21	74 (15.9)
	22	30 (6.4)
	23	8 (1.7)
**Grade**		
	First year	214 (45.9)
	Sophomore	83 (17.8)
	Junior	75 (16.1)
	Senior	82 (17.6)
	Five grade	10 (2.1)
	Six grade	2 (0.4)

**Table 2:** Mental health questionnaire results.

Gender	Nondepression		Depression						
				Mild		Moderate		Severe	
	n (%)	ASR^a^		n (%)	ASR	n (%)	ASR	n (%)	ASR
Female	94 (66.20)	–2.8		34 (23.94)	3.0	9 (6.34)	–0.1	5 (3.52)	0.9
Male	254 (78.40)	2.8		42 (13.00)	–3.0	21 (6.48)	0.1	7 (2.16)	–0.9

^a^ASR: adjusted standardized residual.

**Table 3:** Mann-Whitney U test results.

Variables	Mann-Whitney *U*	Z-score	Asymptotic significance (2-tailed)	Mean value of nondepressive group	Mean value of depressive group
Number of breakfasts	17692.00	–2.25	0.025	52.77	45.81
SD of time of breakfast	16089.00	–2.91	0.004	0.70	0.77
Number of lunches	17071.50	–2.74	0.006	71.93	64.76
Number of lunches during festival^a^	16491.00	–3.22	0.001	4.30	3.23
Lunch Time	16806.00	–2.95	0.003	11.75	11.80
Dinner Time	17445.00	–2.44	0.015	17.47	17.60
Number of dinners	17661.50	–2.27	0.023	65.54	59.25
*TOP*_1_	17479.00	–2.42	0.016	47.59	42.26
*TOP*_2_	17295.00	–2.56	0.010	44.48	39.36
*TOP*_3_	17191.50	–2.64	0.008	42.97	38.13
*TOP*_4_	17234.50	–2.61	0.009	41.97	37.27
*TOP*_5_	17304.00	–2.55	0.011	41.97	37.27
8-9 R^b^	17690.50	–2.31	0.046	3.26	1.94
9-10 R	16063.50	–3.56	0.050	4.80	3.11
10-11 R	17923.00	–2.09	<0.001	3.31	2.36
10-11 O^c^	17450.00	–2.46	<0.001	3.04	3.60
11-12 R	18334.50	–1.99	<0.001	0.80	0.52
13-14 O	17771.00	–2.20	0.008	4.08	4.85
14-15 R festival	18174.50	–2.36	0.012	0.35	0.52
19-20 R festival	17864.00	–2.34	0.014	0.69	1.03
20-21 R	18010.00	–2.00	0.018	7.90	8.89
21-22 R	17352.50	–2.52	0.019	8.28	9.75
22-23 R	14948.50	–4.43	0.021	6.32	9.77
23-24 R	15342.00	–4.25	0.028	2.22	4.36
23-24 O	17501.50	–2.64	0.037	1.72	2.47

^a^Features unlabeled with festival refer to nonfestival days.

^b^R: return to the dormitory.

^c^O: leave the dormitory.

### Behavioral Differences Between the Two Groups of Students

Eating behavior was found to be different between the two groups of students: depressive students (*u*=45.81) skipped breakfasts significantly more often than nondepressive (*u*=52.77) students (*z*=–2.25, *P*=.03). The same was observed for lunch and dinner. In addition, the breakfast time of depressive students was later than that of nondepressive students (*z*=–2.91, *P*=.004), and lunch and dinner time were also later.

With regard to daily schedule preferences, the number of depressive students entering the dorm in the morning was significantly less than that of nondepressed students. In addition, the night activity of depressed students (ie, the average number entering or leaving the dorm between 8 pm and midnight was significantly busier than that of nondepressive students.

**Figure 4:**
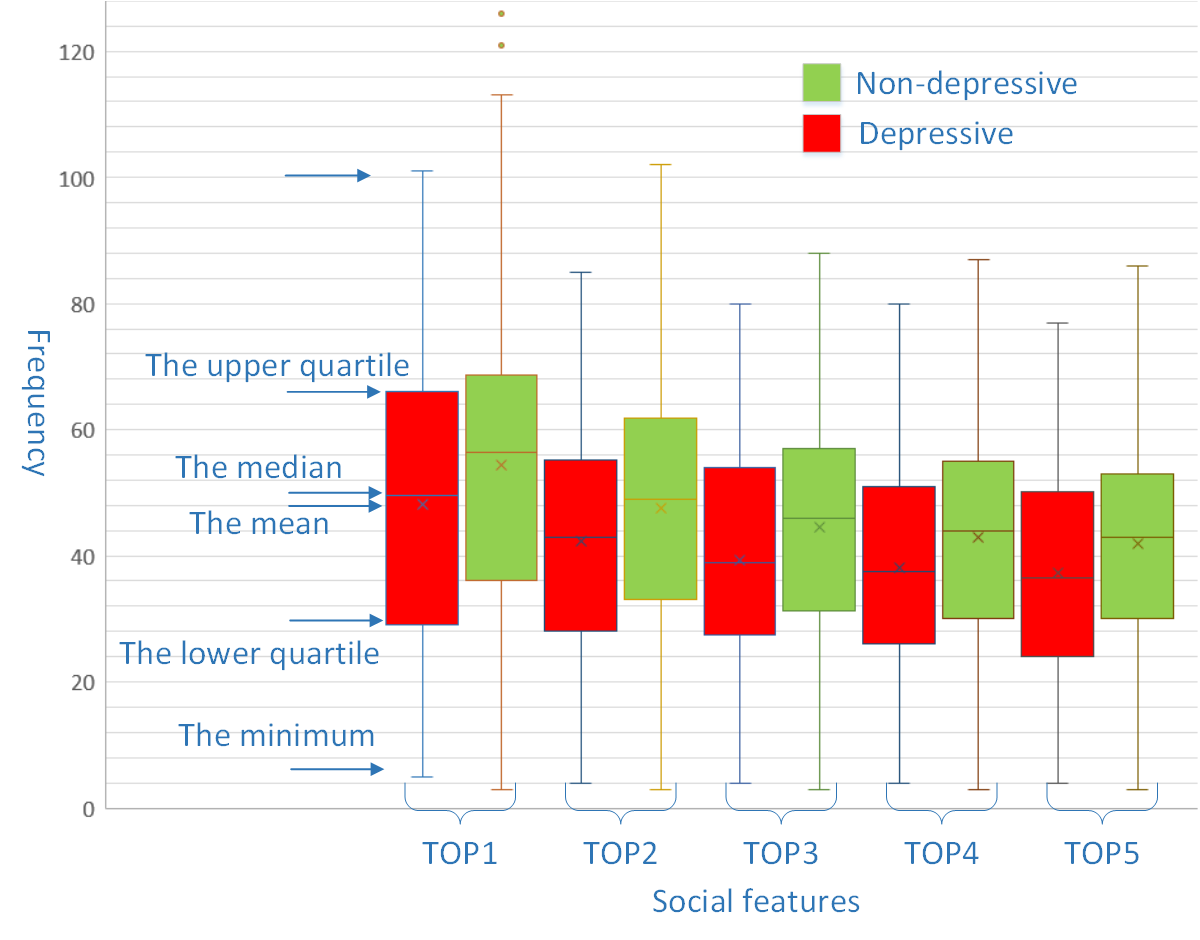
The distribution of social frequency of depressive and non-depressive students.

Regarding social interaction, all 5 social features of depressive students were significantly lower than their counterparts. Figure 4 illustrates the distribution of social behavior features of the two groups. The box plot indicates that the lower quartile, average, median, upper quartile and maximum of the depressive group are all lower than nondepressed students. It is, in turn, revealed that the student card–based social relationship features proposed in this paper can reflect students’ social characteristics.

The number of poverty subsidy applications of depressive and nondepressive students was statistically different (χ2=4.2, *P*=.04), with the application rate of poverty subsidy in the nondepressed group 4.90% and depressed group 10.17%, suggesting that there is a higher rate of depression among economically disadvantaged students.

### Factor and Correlation Analyses

Correlation analysis showed that there was a high correlation between the vast majority of items on the SDS, which means it is possible to use a few factors to summarize most of the information contained in the original 20 entries. Principal component factoring with promax (oblique) rotation was adopted to evaluate the factor structure of the SDS in this study. The Bartlett test of sphericity was significant (χ2=2505.6, *P*<.001), and the Kaiser-Meyer-Olkin measure of sampling adequacy was 0.92, indicating the rationality of principal component factoring.

The eigenvalues of the first 4 principal components were greater than 1, and the variations were 30.15%, 8.01%, 5.72%, and 5.34%, respectively. The cumulative variance was 49.23%. The rotated factor pattern matrix is given in Table 4.

The first factor (F1) was composed of 10 items: decreased libido, personal devaluation, psychomotor retardation, dissatisfaction, decreased appetite, confusion, hopelessness, emptiness, indecisiveness, suicidal ideation. The second factor (F2) was composed of 6 items: crying spells, irritability, fatigue, sleep disturbance, psychomotor agitation, and depressed affect. Factor III (F3) consisted of 3 items: constipation, weight loss, tachycardia. Factor IV (F4) consisted of only one item: worse in the morning. Unlike factor analysis results of some literature [40,48,49], diurnal variation (worse in the morning) was the only item that has large loading in F4. We summed up the scores of all items belonging to each factor or symptom group as the score or severity of the symptom.

**Table 4:** Promax rotated factor pattern matrix (N=466).

Factor and item		F1	F2	F3	F4
**Factor I: cognitive (F1)**					
	Decreased libido (6)	0.731	0.263	–0.032	0.040
	Personal devaluation (17)	0.681	0.029	0.044	0.312
	Psychomotor retardation (12)	0.678	–0.058	0.112	–0.171
	Dissatisfaction (20)	0.651	–0.049	–0.089	–0.021
	Decreased appetite (5)	0.624	0.088	–0.208	–0.424
	Confusion (11)	0.615	–0.132	–0.067	–0.189
	Hopelessness (14)	0.609	–0.105	0.030	0.221
	Emptiness (18)	0.589	–0.156	0.132	0.312
	Indecisiveness (16)	0.583	–0.054	0.090	0.069
	Suicidal ideation (19)	–0.503	0.056	0.087	0.033
**Factor II: manifest depressed mood (F2)**					
	Crying spells (3)	0.125	0.727	–0.057	–0.043
	Irritability (15)	0.015	0.648	0.063	0.174
	Fatigue (10)	0.004	0.640	0.161	–0.057
	Sleep disturbance	–0.049	0.415	0.211	–0.006
	Psychomotor agitation (13)	–0.054	0.754	–0.074	0.056
	Depressed affect (1)	–0.165	0.674	–0.112	–0.052
**Factor III: somatic 1 (F3)**					
	Constipation (8)	–0.022	–0.125	0.820	–0.359
	Weight loss (7)	–0.029	–0.016	0.549	0.175
	Tachycardia (9)	0.139	0.379	0.587	–0.054
**Factor IV: somatic 2 (F4)**					
	Worse in the morning (2)	0.074	0.045	–0.135	0.730

### Daily Routine

Table 5 shows the Spearman correlation between daily routine features and the four factors and the total SDS score.

Among the 4 factors, F4 can be reflected in daily routine most, and the reason for the correlation may be that depression disrupted circadian rhythms [50]. Students with higher levels of depressed mood (F2) and somatic (F3, F4) depressed symptoms partially result in increased night activity, while a decrease in morning activity is partially caused by cognitive (F1), depressive mood (F2), and somatic (F4) symptoms (see Multimedia Appendix 1 for detailed information).

**Table 5:** Spearman correlation between the factors and daily routine.

Scores	6-7 R^a^	7-8 R	7-8 O^b^	8-9 R	8-9 O	9-10 O	23-0 R	23-0 O	0-1 O
F1	.03	–.01	–.109^c^	–.06	–.07	–.125^d^	.05	.07	–.01
F2	.08	.03	–.07	–.07	–.08	–.162^d^	.129^d^	.188^d^	.128^d^
F3	.096^c^	.00	–.01	–.04	–.05	–.05	–.04	.092^c^	.102^c^
F4	–.172^d^	–.179^d^	–.119^d^	–.201^d^	–.165^d^	–.162^c^	.128^d^	.098^c^	.09
SDS^e^	.06	–.01	–.111^c^	–.096^c^	–.105^c^	–.170^d^	.07	.134^d^	.07

^a^R: return to the dormitory.

^b^O: leave the dormitory.

^c^Correlation is significant at the .05 level (2-tailed).

^d^Correlation is significant at the .01 level (2-tailed).

^e^SDS: total Zung Self-Rating Depression Scale score.

### Meal Habit

Depression symptoms can result in changes in appetite [29]. Intuitively, these changes may be reflected in the dining data. To test this hypothesis, we performed the Spearman rank correlation analysis between meal habits and depression symptoms, and the test results are shown in Table 6.

As can be seen from the table, F4 correlates with most meal habit features with high confidence. Feeling worse in the morning can lead to poor meal habits, such as a high rate of skipping meals and irregular eating.

**Table 6:** Spearman correlation between the factors and meal habits.

Scores	SD breakfast time	Mean breakfast time	Number of breakfasts	Number of lunches	Number of dinners
F1	.066	.007	–.053	–.064	–.050
F2	.166^b^	.035	–.083	–.078	–.037
F3	.115^a^	.054	–.014	–.036	–.021
F4	.153^b^	.161^b^	–.275^b^	–.151^b^	–.135^b^
SDS^c^	.129^b^	.038	–.100^a^	–.086	–.064

^a^Correlation is significant at the .05 level (2-tailed).

^b^Correlation is significant at the .01 level (2-tailed).

^c^SDS: total Zung Self-Rating Depression Scale score.

### Academic Performance

As most students should accomplish the majority of courses in the first two years of college life, first-year students are empirically found to be more serious about their study compared with higher year students, therefore we only use first-year student data (214 students) to investigate associations between academic performance and the severity of depression. The result of Spearman correlation is reported in Table 7.

Compared with other factors, F1 has the greatest impact on performance, especially for the two extremes, fail and excellent. When the F1 score is high, it is easier to fail exams or to get general grades. However, the total SDS score was only found to significantly correlate with the fail course rate and the number of failed courses. The table suggests that depression makes it easier for students to have bad academic performance, even if the total score does not reach the threshold of depression.

**Table 7:** Spearman correlation between depression and academic performance.

Scores	Excellent rate	Fail rate	Number of excellent	Number of good	Number of fail
F1	–.158^a^	.185^b^	–.137^a^	.157^a^	.186^b^
F2	.061	.150^a^	–.043	.071	.155^a^
F3	.108	.090	.117	–.061	.097
F4	–.050	.132	–.039	.018	.140^a^
SDS^c^	–.111	.195^b^	–.087	.116	.201^b^

^a^Correlation is significant at the .05 level (2-tailed).

^b^Correlation is significant at the .01 level (2-tailed).

^c^SDS: total Zung Self-Rating Depression Scale score.

### Social Behavior

The distribution of social behavior features is normally distributed, so we tested the Pearson correlation between features and 4 facts and SDS score. The result is shown in Table 8.

The table indicates that F4 had the largest effect on social activity, students who felt bad in the morning were more reluctant to share meal times with their friends, while the total SDS score only has a weak relationship with social activity.

The relationship between the frequency of library access and depression was also explored, F4 was found to be negatively correlated with the number of library visits (*r*=–.179, *P*<.001). No difference was found in the acquisition of scholarships.

**Table 8:** Pearson correlation between social features and depression.

Scores	*TOP*_1_	*TOP*_2_	*TOP*_3_	*TOP*_4_	*TOP*_5_
F1	–.053	–.064	–.064	–.055	–.054
F2	–.094^a^	–.096^a^	–.093^a^	–.084	–.083
F3	–.059	–.056	–.050	–.037	–.042
F4	–.166^b^	–.156^b^	–.164^b^	–.171^b^	–.166^g^
SDS^c^	–.096^a^	–.102^a^	–.101^a^	–.091^a^	–.090

^a^Correlation is significant at the .05 level (2-tailed).

^b^Correlation is significant at the .01 level (2-tailed).

^c^SDS: total Zung Self-Rating Depression Scale score.

## Discussion

### Principal Findings

Being a serious mental disorder, depression affects the life of patients in both mental and physical aspects. There is a critical need to identify behavior characteristics for better identifying and even helping the treatment of depression; however, to the best of our knowledge, research on depression among college students with campus data has not been conducted.

The meal habits of depressive students in this research were different from that of nondepressive students; this finding is congruent with the finding of Lee et al [29] that implied depression may affect the dietary habits. Our experiments indicated students with depression tended to have bad eating habits. Factor analyses indicated feeling bad in the morning was a contributor to the phenomena; furthermore, irregular breakfast time was caused by complicated symptoms except for cognitive factor.

Depressed students were found to have fewer morning activities and more night activities; furthermore, factor analyses indicated that decreased morning activity was affected by factor 4 (feeling bad in the morning), while increased evening activity was partially caused by factor 2 (depressed mood) compared with other symptoms.

Our findings on daily routine and meal habits are consistent with the well-established literature that suggests biologic rhythm is associated with depression [35-38,51-53]. Disruptions in behavioral patterns caused by depression during waking hours includes not only the volume of activity but also the patterns of behavior [53,54]. These pattern changes may be a result of the genetic and hormonal factors [36] implicated in depression-related circadian rhythm changes [53,54].

With respect to social interaction pattern, the Mann-Whitney *U* test and correlation analyses demonstrated that depression impaired social activity, and this conclusion is congruent with previous studies [38,55]. Furthermore, our study demonstrated that social activity extracted in this research was mainly affected by diurnal variation (F4), while cognitive symptoms (F1) and somatic symptoms (F3) had no connection with decreased social activity.

Many studies have investigated the negative impact of depression on academic performance [5,26,56], and our study goes further on this topic. Factor analyses demonstrated that there was a negative correlation between depression and course fail rate among first-year students. Moreover, by investigating the relationship between academic performance and each factor, we found that the cognitive factor (F1) was the most relevant factor for poor academic performance. Students with high cognitive symptom were more likely to fail exams and less likely to achieve excellent academic performance [57].

Finally, through the chi-square test we found that the proportion of poverty subsidy application was not the same between depressive and nondepressive students. The application proportion of the depressive group was significantly higher than the nondepressive group, which indicates that family economic status is one of the impact factors in depression. This conclusion fits into previous studies, which demonstrated poor socioeconomic background is associated with depressive symptoms within each country [5,56].

### Limitations

Although our research reveals that some behavioral characteristics of depressive students can be captured by campus data, the results are preliminary and a number of limitations must be mentioned.

First, the work has not covered all aspects of students’ life on campus (eg, data related to location, physical exercise, after school clubs, course selection) due to the limitation of school information infrastructure. With respect to meal habits, because there are other places for students to eat except for the campus canteen, it is impossible to accurately obtain all the dining information. Thus, the term meal habits described in the paper only represents the meal habits in the campus canteen.

Second, although some patterns about depressive students’ daily routines were found, specific reasons need to be further clarified through a questionnaire survey.

Third, this research adopted the method of self-reported questionnaire survey to determine whether participants were depressed, which may bring about the pervasive problem of response bias [58].

Finally, students with different levels of depression may behave differently. Because of the low number of students who suffered from moderate to severe depression, behavioral differences among mild, moderate, and severe depression were not explored.

### Implications and Future Work

This paper proves that campus data can reflect student depression status and indicates that depression may be predicted by using machine learning techniques. Because timely rescue of potentially depressed students can improve their academic performance and reduce their own pain and suicide risk, depression prediction has good practical effects.

Privacy presents a major problem. To precisely predict students’ mental health problems, too much privacy data such as online behavior, daily routine, family background, and consumption level may be involved. Thus, it is recommended that data providers convert or add noise to the original data to protect privacy. For example, provide researchers with differential privacy [59] processed data.

In addition, data-driven mental health prediction methods can lead to some negative effects, and the feedback loop is a topic worthy of consideration because it creates some problems.

The principle of educational equity may be violated. Managers may prefer not to grant scholarships to depressed students because they may perform poorly, and this would reduce their chances of participating in academic, community, and competition activities. This could be addressed by developing a mental health prediction system that can only be used by those whose jobs are related to mental health care. When the system finds a depressed student, a psychological counselor will contact the student directly to decide whether it is appropriate to recommend that they ask for psychological counseling services according to the actual situation. Because of direct communication between the counselor and depressed student, no other students or teachers would know the specific situation, therefore eliminating the problem of educational inequality that may be caused by depression.

Although data management rights belong to the information technology department, it does not mean that the department has full rights to the data. Some operations on the data are forbidden to perform unless permitted by students. Some students may be concerned about mental health issues or personal privacy being exposed, which may result in a refusal of authorization. Therefore, some depressive students will not be detected by the system. To address this, we will popularize mental health knowledge on campus to eliminate student stigma about mental illness and explain to students how the system works to eliminate their concerns about privacy.

### Conclusions

The campus information systems run all the time and record all aspects of students’ living on campus. Compared with the traditional questionnaire survey method, these data can reflect behavior and psychological activities more objectively and realistically, and there is almost no cost to obtain this data compared with other types of data (eg, mobile phone use) [54]. This paper proves that depressive students have different behavioral characteristics than nondepressive students by using campus data and the symptoms of depression can also be reflected in the data, supporting scholars’ behavioral models of depression [24]. This paper also provides new ideas for discovering human behavior patterns associated with depression and other mental health disorders. If combined with other types of campus data (eg, network connection event logs [60]), it is possible to achieve an artificial intelligence–based student depression prediction method.

## Appendix

Multimedia Appendix 1 Supplementary material for Table 5.

## Abbreviations

BDI: Beck Depression Inventory
ISA: information system administrator
OR: odds ratio
SDS: Zung Self-Rating Depression Scale
